# Long-term consequences of *in utero* irradiated mice indicate proteomic changes in synaptic plasticity related signalling

**DOI:** 10.1186/s12953-015-0083-4

**Published:** 2015-11-16

**Authors:** Stefan J. Kempf, Christine von Toerne, Stefanie M. Hauck, Michael J. Atkinson, Mohammed A. Benotmane, Soile Tapio

**Affiliations:** Institute of Radiation Biology, Helmholtz Zentrum München, German Research Center for Environmental Health GmbH, Ingolstaedter Landstrasse 1, 85764 Neuherberg, Germany; Research Unit Protein Science, Helmholtz Zentrum München, German Research Center for Environmental Health GmbH, Neuherberg, Germany; Chair of Radiation Biology, Technical University Munich, Munich, Germany; Radiobiology Unit, Belgian Nuclear Research Centre, SCK-CEN, Mol, Belgium; Present address: Department of Biochemistry and Molecular Biology, University of Southern Denmark, Odense, Denmark

**Keywords:** Learning, Memory, Ionising radiation, CREB, *in utero* irradiation, Hippocampus, Cortex, Proteomics

## Abstract

**Background:**

The harmful consequences of *in utero* irradiation on learning and memory have been recognised but the molecular mechanisms behind the damage are still unknown.

**Results:**

Using a mass spectrometry-based approach, we investigated the long-term changes in the global cortical and hippocampal proteome 6 months after 0.1, 0.5 and 1.0 Gy *in utero* X-ray irradiation delivered on embryonic day 11 in male C57Bl/6 J offspring. We noted alterations in several signalling pathways involved in cognition, the transcription factor cAMP response element-binding protein (CREB) playing a central role. Immunoblotting of CREB and phosphorylated CREB (Ser133) showed an altered expression profile at all doses in the hippocampus and at 0.5 and 1.0 Gy in the cortex. The greatest reduction in the phospho-CREB level was seen at 1.0 Gy in the hippocampus. It was accompanied by enhanced expression of postsynaptic density protein 95 (PSD95), suggesting effect on synaptic plasticity in neuronal dendrites.

**Conclusions:**

As the CREB signalling pathway plays a crucial role in neuronal plasticity and long-term memory formation in the brain, the radiation-induced alterations of this pathway seen here are in good agreement with the cognitive dysfunction seen in *in utero* irradiated populations. These data contribute to a deeper biological understanding of molecular mechanisms behind the long-term damage induced by relatively low doses of ionising radiation during gestation.

**Electronic supplementary material:**

The online version of this article (doi:10.1186/s12953-015-0083-4) contains supplementary material, which is available to authorized users.

## Background

Epidemiological studies on atomic bomb survivors exposed *in utero* show that ionising radiation may induce permanent cognitive dysfunction, particularly if the exposure occurred during early gestational phases (weeks 8 to 15) [1]. This developmental phase of the brain is characterised by rapidly dividing cells called the brain growth spurt [2]. It is probable that ionising radiation during the early gestational phases targets mainly proliferating and differentiating cells in the brain [1, 3]. In addition to cortex, the hippocampus is important in learning and memory [4, 5] and shows a high degree of proliferation and lifelong neurogenesis where neural stem cells give rise to neural progenitor cells and mature neurons. This process is known to be negatively affected by irradiation [6–9]. Thus, not only the cortex, but also the hippocampus may be a target for *in utero* irradiation damage.

The early gestational weeks 7 and 8 in human relate to embryonic days E11 and E12 in mice [10]. This time point has been shown to be the most radiation-sensitive period in mural gestation when doses as low as 0.35 Gy cause significant reduction in locomotor and exploratory activities [11]. This period coincides with the earliest time point for generation of neurons in the cerebral cortex of *Mus musculus* [12, 13].

We have recently demonstrated that both male NMRI and female C57BL/6J mice irradiated at postnatal day 10 (PND10) with radiation doses as low as 0.5 Gy show changes in synaptic signalling pathways several months post-irradiation [14, 15]. Especially the long-term potentiation/depression-associated CREB signalling was shown to be altered in whole hippocampus and cortex [14] as well as synaptosomes isolated from these [15]. In agreement with this, both male and female C57BL/6J mice as well as male NMRI mice showed cognitive behaviour deficits after irradiation with 0.5 Gy but not at lower doses [16], (Per Eriksson, personal communication).

The aim of this work was to elucidate the molecular consequences, especially those in the CREB signalling pathway, observed in adult mice after *in utero* irradiation. Pregnant C57Bl/6J mice were irradiated on E11 with total body doses of 0.1, 0.5 and 1.0 Gy. Alterations in cognitive signalling pathways of the hippocampus and cortex were investigated in the male offspring 6 month post-irradiation using mass spectrometry- and immunoblotting-based experimental approaches.

## Results and Discussion

### *In utero* irradiation affects long-term hippocampal and cortical signalling pathways associated with CREB-mediated synaptic plasticity

**Fig. 1:**
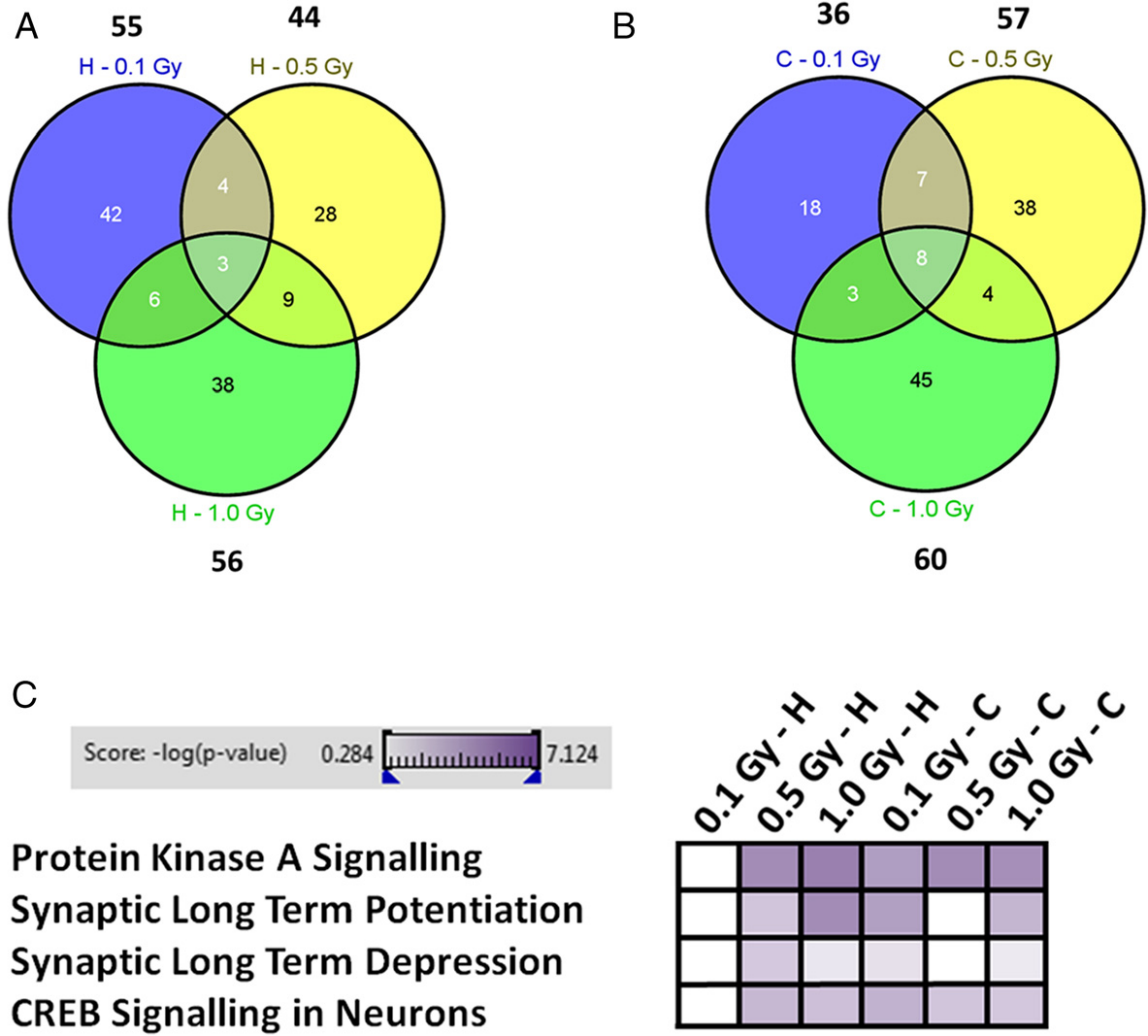
Mass spectrometry-based proteomics of hippocampus and cortex of *in utero* irradiated animals. Venn diagrams show the number of all and shared deregulated proteins in hippocampus (**a**) and cortex (**b**) exposed to 0.1, 0.5 and 1.0 Gy 6 months after *in utero* irradiation using global proteomics approach; *n* = 3. The comparison of proteomic profiles was performed on sham-irradiated control mice of the same age. The number above each dose represents the total number of deregulated proteins found at this dose. Altered learning- and memory-related signalling pathways at all doses using the Ingenuity Pathway Analysis software are shown (**c**). The high colour intensity indicates high significance (p-value) of the pathway; all coloured boxes have a *p*-value of ≤ 0.05 whereas white boxes have a *p*-value of > 0.05 and are thus not significantly altered. H: hippocampus; C: cortex

Mass spectrometry-based global proteomics using Isotope Coded Protein Label method (ICPL quadruplex approach) showed a deregulation of several proteins in the total hippocampus (0.1 Gy/0.5 Gy/1.0 Gy: 55/44/56 proteins) and entire cortex (0.1 Gy/0.5 Gy/1.0 Gy: 36/57/60 proteins) 6 months post-irradiation (Fig. 1 a and b). The number of deregulated proteins address 11 %/9 %/12 % in the hippocampus and 12 %/19 %/21 % in the cortex at radiation doses of 0.1, 0.5 and 1.0 Gy, respectively, in relation to the total number of confident quantifiable proteins (≥2 unique ICPL-labelled peptides found in n-1 runs, ICPL variability ≤ 30 %).

Importantly, only a few proteins were shared between all the applied doses in the same brain region (Fig. 1 a and b) or between the brain regions at similar doses (Additional file 1: Figure S1 A, B and C). However, the majority of shared proteins were deregulated in the same direction (SI Additional file 2: Table S2, Additional file 1: Figure S1 D, E and F). SI Additional file 2: Table S1 shows the list of identified deregulated proteins categorised by radiation dose.

The expression of several (serine/threonine) protein kinases, protein phosphatases and mitochondrial respiratory proteins (SI Additional file 2: Table S1) was found to be altered by irradiation. In the hippocampus, we found at 0.1, 0.5 and 1.0 Gy 1/3/3 (serine/threonine) protein kinases, 3/3/2 protein phosphatases and 5/3/4 mitochondrial respiratory proteins, respectively. Similarly, in the cortex at 0.1, 0.5 and 1.0 Gy a change in 4/2/5 (serine/threonine) protein kinases, 1/1/4 protein phosphatases and 3/10/6 mitochondrial respiratory proteins, respectively, was found. Thus, no dose-dependent increase in the number of proteins in these groups or in the total number of deregulated proteins can be seen. This result is in agreement with our previous studies with neonatally irradiated mice where the persistent changes in the amount of deregulated proteins showed no dose-dependency, except with the very low dose (0.02 Gy) where the number of deregulated proteins was smaller than at higher doses [14, 15].

A significant increase in redox scavengers such as peroxiredoxin (Prdx5) at 0.1 and 0.5 Gy in the hippocampus and superoxide dismutase (Sod1) at 1.0 Gy in the cortex was observed (SI Additional file 2: Table S1). Similar persistent increase in the level of Prdx5 has been found previously in the hippocampus and cortex of neonatally irradiated mice [14, 15]. The increase in the levels of radical scavengers may reflect enhanced amounts of reactive oxygen species produced by altered mitochondrial respiratory complexes [17]. However, analysis of radiation-induced changes in the mitochondrial respiration as previously seen in hippocampal mitochondria after neonatal irradiation [15] is necessary to verify this. Interestingly, the level of ubiquinol-cytochrome c reductase core protein 1 (Uqcrc1), a member of the respiratory Complex III, was increased after *in utero* irradiation at all doses in the hippocampus and cortex (SI Additional file 2: Table S1, SI Additional file 2: Table S2). The expression of this protein was also found increased in hippocampus and cortex of neonatally irradiated NMRI and C57BL/6 mice several months post-irradiation [14, 15]. Overexpression of Uqcrc1 has been associated with neurological symptoms in mice, coupled to enhanced complex III activity in neuroblastoma cells [18].

In this study, a decrease in the level of the Rac1 protein was observed in the cortex at 1.0 Gy. Rac1 is a small RhoGTPase that regulates actin polymerisation in the synapse [19]. Radiation-induced decrease in Rac1 expression has been found frequently in hippocampus and cortex [15, 16, 20] implying its essential role at early developmental stages.

Increased levels of ankyrin2 (Ank2) were found in this study at 0.1 Gy in both hippocampus and cortex. Similar upregulation of this protein was identified previously in hippocampus [14] and cortex [15] of neonatally irradiated mice. Ank2 is necessary to elevate cyclic AMP (cAMP) levels in neurons [21]. Importantly, we found a number of changes in cAMP-dependent regulatory protein kinases in the cortex at 0.1 Gy (increase in Prkacb level), 0.5 Gy (decrease in Prkacb level) and 1.0 Gy (increase in Prkar2a level) (SI Additional file 2: Table S1). The cAMP levels influence the activity of protein kinases A (Prka’s) and mitogen-associated protein kinases (MAPK’s) that regulate phosphorylation of cAMP-responsive element binding protein (CREB) on Ser133 [22]. Moreover, calmodulin protein kinases (CaMK’s) are also involved in the process of CREB phosphorylation [22, 23]. The expression levels of Prka’s and CaMKs were altered in the hippocampus (0.5 Gy, 1.0 Gy). In cortex, these proteins were found to be deregulated at all doses (Additional file 2: Table S1 – green and orange highlighted PANTHER protein classes). Both types of kinases are essential in learning and memory formation [24, 25]. Interestingly, the protein kinase Map2k1 that is also involved in the regulation of the CREB activity was increased in both hippocampus and cortex only at 0.1 Gy (Additional file 2: Table S1—brown highlighted PANTHER protein class).

These data are consistent with the outcome of the pathway analysis showing that radiation-affected signalling pathways related to memory formation (PKA signalling, synaptic long-term potentiation and depression, CREB signalling) are affected at all radiation doses in the cortex but only at 0.5 and 1.0 Gy in the hippocampus (Fig. 1 c). These pathways are interconnected as PKA is involved in the maintenance of synaptic long-term potentiation [26, 27] through activation of the transcription factor CREB [28]. CREB functions as the common downstream transcription factor for all these pathways.

### *In utero* irradiation alters CREB signalling in adult mice

**Fig. 2:**
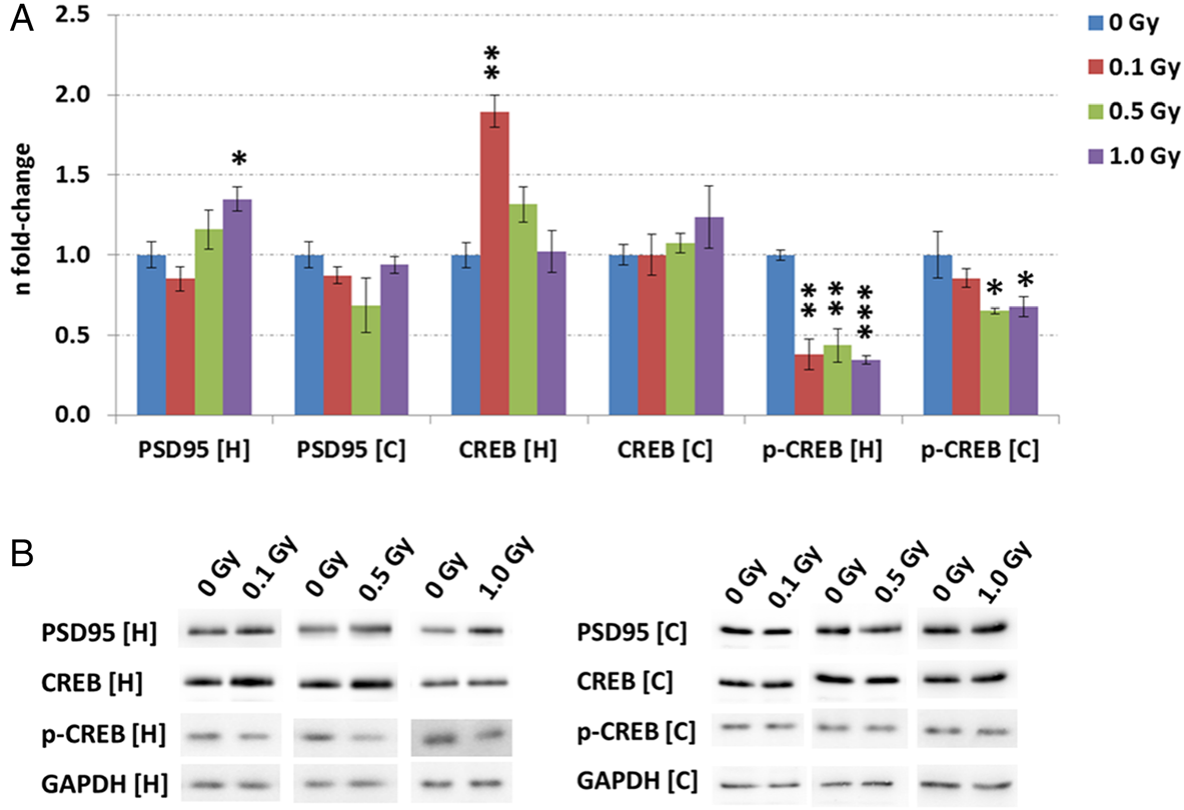
Analysis of CREB, phospho-CREB and PSD95 levels using immunoblotting in cortex and hippocampus. Columns representing the fold-changes with standard errors of the mean (SEM) of relative protein expression of CREB, phospho-CREB and PSD95 in control (sham-irradiated) and irradiated mouse brains at same age are shown (**a**); *n* = 3. Immunoblot verification of the protein expression is shown (**b**). *p < 0.05; **p < 0.01; ***p < 0.001 (unpaired Student’s *t*-test). Normalisation was performed against endogenous GAPDH. H: hippocampus; C: cortex

To further elucidate the putative radiation-induced changes in the CREB signalling pathway, we quantified total CREB and phosphorylated CREB (Ser133) levels from total homogenates from whole hippocampus and cortex at all doses. Generally, an inhibition of CREB phosphorylation interferes with synaptic plasticity and recognition memory in cortex and hippocampus [29, 30]. We noted no change in total CREB levels but a decrease in phospho-CREB at 0.5 and 1.0 Gy in the cortex (Fig. 2 a and b). In the hippocampus, an increased level of total CREB was observed only at 0.1 Gy. The level of phosphorylated CREB was decreased at all doses (Fig. 2 a and b). As we did not note any significant alterations in CREB-related pathways at 0.1 Gy in the hippocampus (Fig. 1 c) we suggest that the decreased level of phospho-CREB is compensated by the increased total level of CREB and thus no substantial hippocampus-dependent effect on memory and learning is seen at this dose. It has been shown previously that the elevation of CREB expression alone is sufficient to increase hippocampus-dependent memory in hippocampal pyramidal neurons [31–33]. In accordance with this, we have previously shown that the dose of 0.1 Gy given on PND10 had no long-term effect on the cognitive behaviour in contrast to higher doses in NMRI mice [14]. Recently, it was shown that the same strain of male mice as used in this study that were irradiated *in utero* with doses of 0.5 and 1.0 Gy had a reduced hippocampal-dependent spatial learning and memory capacity at the age of 12 weeks, as evaluated by different behavioural tests [34]. Further, it was demonstrated that the animals irradiated with 1.0 Gy at E11 showed at the age of 40 weeks depletion of the neuronal marker N-acetyl-aspartate in the cortex, indicating neuronal cell loss accompanied with enlarged ventricles and impaired juvenile hippocampal neurogenesis [34]. Consequently, the molecular changes observed here in the CREB signalling may partially reflect the difference in cellular composition between sham-irradiated and irradiated brain.

Interestingly, the most significant reduction in the level of phosphorylated CREB was noted at 1.0 Gy in the hippocampus. This was coinciding with an increase of the PSD95—a protein exclusively localised in neuronal dendrites [35] (Fig. 2 a and b). This was in accordance with our proteomics data (PSD95: fold-change of 1.41) (Additional file 2: Table S1). The establishment of learning depends on the strength and number of synaptic connections and alterations in PSD95 expression levels are able to affect this synaptic plasticity process. Previous studies have shown that a deletion in the PSD95 protein leads to imbalances between long-term potentiation and long-term depression through altered function of glutamatergic NMDA receptors [36]. This enhances long-term potentiation status that is accompanied by severely impaired spatial learning [37]. In contrast, PSD95 overexpression elevates the ratio of excitatory-to-inhibitory synaptic contacts [37] leading to impairment in neurotransmission, thus also affecting the memory function. The increased expression of this protein in our study is consistent with recently published data showing increased PSD95 levels in neurons of the hippocampal granule cell layer 1 month after postnatal radiation exposure (1.0 Gy) [38]. Further, our data are in good agreement with our previous studies demonstrating that acute ionising radiation affects synaptic plasticity in vitro and in vivo at 1.0 Gy [16].

## Conclusions

To the best of our knowledge, this study provides the first attempt to perform global proteomics analysis of murine hippocampus and cortex several months after a prenatal exposure to ionising radiation. We show that gestational irradiation even at relatively low doses leads to persisting alterations in these brain regions including changes in the memory-related CREB signalling. This result is in good agreement with the cognitive dysfunction observed in *in utero* exposed populations. The molecular similarity of these data to our previous results with male and female NMRI and C57BL/6J strains may be due to the very early age of irradiation (PND10 and E11); the mice had not reached puberty where the sexual hormones may start influencing the outcome. However, further studies with bigger number of animals are important to clarify this point. Our data contribute to a deeper biological understanding of the adverse effects of ionising radiation on the developing brain.

## Methods

### Ethics statement, irradiation of animals and tissue collection

Experiments were carried out in accordance with the European Communities Council Directive of November 24, 1986 (86/609/EEC) and approved by the local ethical committee SCK-CEN/VITO (ref. 02–012). Pregnant C57Bl/6J mice (Janvier - Bio-services, Uden, The Netherlands) were total body irradiated on embryonic day 11 (E11) of gestation with a single exposure to X-ray irradiation (0.35 Gy/min, Pantak RX tube operating at 250 kV, 15 mA [1 mm Cu-filtered X-rays]) at doses of 0 (sham-irradiated control), 0.1, 0.5 and 1.0 Gy (Radiobiology Unit, Belgian Nuclear Research Centre, SCK-CEN, Mol, Belgium). Dose verification was done with an ionisation chamber. The sham-irradiated and irradiated male offspring from comparable litter sizes were kept until 6 months post-irradiation and sacrificed via cervical dislocation. Brains were excised and transferred to ice-cold phosphate buffered saline (PBS), rinsed carefully, and dissected under stereomicroscopic inspection under cold conditions. Entire hippocampi and cortices without meninges from each hemisphere were separately sampled, gently rinsed in ice-cold PBS and snap-frozen in liquid nitrogen. Samples were stored at −80 °C until isolation of proteins. Three independent biological replicates per dose group were tested. These animals originated from 12 different pregnant mice.

### Isolation of total protein

Brain tissues (total hippocampus and cortex) were homogenised in 6 M guanidine hydrochloride on ice using a manual plastic mortar. Homogenates were briefly vortexed, sonicated, and cleared by centrifugation (20,000×g, 1 h, 4 °C). The supernatants were collected and stored at −20 °C before further use. Total protein content was determined using Bradford assay (Thermo Fisher) following the manufacturer’s instructions.

### Mass spectrometry-based proteome analysis

The Isotope Coded Protein Label (ICPL) quadruplex mass spectrometry approach was used to identify differences in the global protein levels in total protein homogenates from hippocampus and cortex as reported in detail elsewhere [16]. Briefly, three individual replicates of whole cortex and hippocampus were used for proteomic analysis at each radiation dose. After reduction and alkylation of Cys-residues, the proteins were labelled with ICPL reagents as follows: control (sham-irradiated) with ICPL-0, 0.1 Gy sample with ICPL-4, 0.5 Gy sample with ICPL-6 and 1.0 Gy sample with ICPL-10. The labelled samples representing each radiation dose per brain region were combined, followed by 1-dimensional protein separation with 12 % SDS-polyacrylamide gel electrophoresis as described elsewhere [16]. Visualisation of protein bands was performed with Coomassie Blue and the gel lanes were cut into five equal slices, destained and overnight trypsinised as described recently [16, 39]. LC-MS/MS analysis was performed on a LTQ-Orbitrap XL device (Thermo Fisher) coupled with a nano-HPLC (Ultimate 3000, Dionex). LC-MS/MS conditions and parameters of analysis are described in detail elsewhere [16, 39]. Briefly, ICPL pairs (ICPL4/ICPL0, ICPL6/ICPL0 and ICPL10/ICPL0) were analysed with Proteome Discoverer software (Version 1.3—Thermo Fisher). To be highly confident in the deregulation of each protein, we applied the percolator algorithm (q < 0.01) and a false discovery rate (FDR) criteria (FDR < 0.01) as used previously [14, 40]. Proteins were considered significantly deregulated if they fulfilled the following criteria: (i) identification by at least two unique peptides in n-1 mass-spectrometry runs (n: number of biological replicates), (ii) quantification with an ICPL-variability of ≤ 30 % and (iii) a fold-change of ≥ 1.3 or ≤ −1.3. The raw-files of the obtained MS-MS spectra can be found under http://storedb.org/project_details.php?projectid=47 with dataset-ID 54 (http://storedb.org/dataset_details.php?do=details&datasetid=54).

### Bioinformatics analysis

A signalling pathway analysis was performed with all deregulated proteins for each dose group using INGENUITY Pathway Analysis (IPA) (http://www.ingenuity.com) applying databases of experimental and predictive origin. Only the database information regarding central nervous system (CNS) was used to be confident about the potentially affected learning- and memory-related signalling pathway changes. Core and comparison signalling pathway analyses were performed with all deregulated proteins from each dose group using IPA. The IPA comparison analysis ranks the significantly affected signalling pathways based on the calculated p-value and reports it hierarchically. The significance values (p-values) between each biological or molecular event and the imported proteins are generated by the software using the Fischer’s exact test. Signalling pathways were reported to be significantly changed if they reached a *p*-value of ≤ 0.05. The software PANTHER (http://www.pantherdb.org) was used to annotate proteins via the PANTHER protein class function.

### Quantification of PSD95, CREB and phospho-CREB levels via immunoblotting

Immunoblotting was performed as recently described [16]. Used primary antibodies are shown in SI Additional file 2: Table S3. The level of GAPDH was not significantly deregulated in any sample based on the global proteomics results and was therefore used as a loading control. In addition, Ponceau S staining was used to evaluate the signal intensity of total protein lysate; only lanes with a similar pattern and intensity of lanes stained with PonceauS were considered for further quantification. Immunoblots were quantified with TotalLab TL100 software (www.totallab.com) using software-suggested background correction. Three biological replicates were used for statistical analysis (unpaired Student’s *t*-test) with a significance threshold of 0.05. These biological replicates (individual mice) were the same as used for the proteomics analysis.

## Additional files

Additional file 1: Figure S1. Mass spectrometry-based proteomics – comparison of overlapping proteins between hippocampus and cortex at the same radiation dose. Venn diagrams showing the number of overlapping proteins between hippocampus and cortex at 0.1 Gy (A), 0.5 Gy (B) and 1.0 Gy (C). The panels D to F show the overlapping proteins between hippocampus and cortex at the different radiation doses with fold-changes, variability and number of counts from the global mass-spectrometry proteomics experiments. D: 0.1 Gy; E: 0.5 Gy; F: 1.0 Gy. (PDF 186 kb) Additional file 2: Table S1. Deregulated proteins found in hippocampus and cortex of *in utero* irradiated mice in comparison to sham-irradiated mice 6 months post-irradiation using mass spectrometry-based proteomics. Complete detailed list of deregulated proteins (name, unique peptides, n-fold-change, variability and counts per biological replicate) obtained from cortex and hippocampus 6 months after *in utero* irradiation at doses of 0.1, 0.5 and 1.0 Gy; *n* = 3. Deregulated proteins were categorised into protein classes using PANTHER classification system software and the general annotation from UniProt as indicated by an asterisk. Green highlighted proteins function as calmodulin/calmodulin protein kinases; orange ones as protein kinases and brown ones as key proteins in MAPK signalling pathway. **Table S2.** Mass spectrometry-based proteomics – comparison of dose-independent overlapping proteins found in hippocampus and cortex. Overlapping deregulated proteins found in hippocampus and cortex at doses of 0.1, 0.5 and 1.0 Gy are shown with protein names and fold changes; *n* = 3. **Table S3.** Antibodies used for immunoblotting. (XLS 138 kb)

## Abbreviations

Gy: Gray
CREB: cAMP response element-binding protein
PSD95: Postsynaptic density protein
CaMK: Calmodulin protein kinase
PKA: Protein kinase A
MAPK: Mitogen-activated protein kinase
PrK: Protein kinase
PBS: Phosphate-buffered saline
ICPL: Isotope coded protein label
HPLC: High performance liquid chromatography
LC-MS/MS: Liquid chromatography tandem mass spectrometry
IPA: Ingenuity pathway analysis
CNS: Central nervous system
GAPDH: Glyceraldehyde 3-phosphate dehydrogenase
