# Supplementary material for: Long-term consequences of in utero irradiated mice indicate proteomic changes in synaptic plasticity related signalling
Source: Proteome Sci. 2015 Nov 16;13:26. doi: 10.1186/s12953-015-0083-4 (PMC4647474; doi:10.1186/s12953-015-0083-4)
Supplement: Additional file 1: Figure S1. — Mass spectrometry-based proteomics – comparison of overlapping proteins between hippocampus and cortex at the same radiation dose. Venn diagrams showing the number of overlapping proteins between hippocampus and cortex at 0.1 Gy (A), 0.5 Gy (B) and 1.0 Gy (C). The panels D to F show the overlapping proteins between hippocampus and cortex at the different radiation doses with fold-changes, variability and number of counts from the global mass-spectrometry proteomics experiments. D: 0.1 Gy; E: 0.5 Gy; F: 1.0 Gy. (PDF 186 kb) [file 12953_2015_83_MOESM1_ESM.pdf]

A

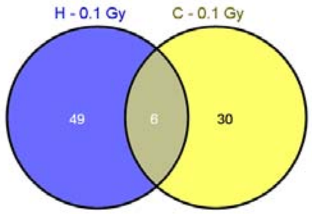

D

0.1 Gy	Hippocampus			Cortex		
Name	fold-change	Variability [%]	counts	fold-change	Variability [%]	counts
Uqcrc1	1.77	19.1	2;2;1	1.69	2.9	2;1;-
Ank2	1.69	28.5	2;2;1	1.53	8.4	1;2;-
Map2k1	1.65	13.6	2;2;1	1.32	3.8	2;-;1
Sfxn1	1.44	22.2	1;2;1	1.80	29.1	1;2;1
Rpl3	1.33	6.2	2;5;1	1.82	18.6	1;1;1
Slc1a3	-1.36	1.9	1;-;3	1.36	17.8	1;-;3

B

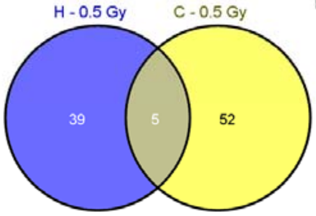

E

0.5 Gy	Hippocampus			Cortex		
Name	fold-change	Variability [%]	counts	fold-change	Variability [%]	counts
Uqcrc1	1.79	23.3	2;1;2	2.22	28.4	2;1;-
Bin1	1.52	27.8	-;2;1	1.60	13.6	3;3;1
Slc3a2	1.47	17.8	-;1;1	1.61	26.1	1;2;-
Sirpa	1.34	27.0	2;1;1	1.41	8.5	2;4;1
Gnb1	1.34	29.6	8;9;8	1.39	4.4	8;5;6

C

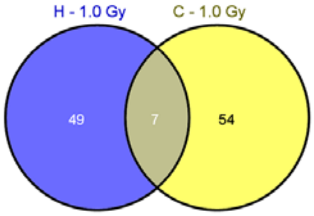

F

1.0 Gy	Hippocampus			Cortex		
Name	fold-change	Variability [%]	counts	fold-change	Variability [%]	counts
Uqcrc1	1.47	16.7	2;2;1	1.95	16.8	2;1;1
Bin1	1.43	3.0	1;2;-	1.48	11.1	3;2;1
Bcas1	1.41	17.0	1;1;-	1.72	18.6	3;2;2
Pygb	-1.31	23.9	10;8;3	-1.32	10.5	3;8;6
Camk2d	-1.31	25.9	7;4;2	1.49	21.8	3;2;2
Atp6v1e1	-1.31	12.2	5;4;7	-1.30	14.8	5;7;5
Cyc1	-1.33	12.6	3;2;4	-1.35	19.5	5;4;2

Figure S1: Mass spectrometry-based proteomics – comparison of overlapping proteins between hippocampus and cortex at the same radiation dose

Venn diagrams showing the number of overlapping proteins between hippocampus and cortex at 0.1 Gy (A), 0.5 Gy (B) and 1.0 Gy (C). The panels D to F show the overlapping proteins between hippocampus and cortex at the different radiation doses with fold-changes, variability and number of counts from the global mass-spectrometry proteomics experiments. D: 0.1 Gy; E: 0.5 Gy; F: 1.0 Gy.
